# Genetic Evaluation of In Vitro Micropropagated and Regenerated Plants of *Cannabis sativa* L. Using SSR Molecular Markers

**DOI:** 10.3390/plants11192569

**Published:** 2022-09-29

**Authors:** Kostas Ioannidis, Ioanna Tomprou, Vangelis Mitsis, Polyxeni Koropouli

**Affiliations:** 1 Laboratory of Sylviculture, Forest Genetics and Biotechnology, Institute of Mediterranean and Forest Ecosystems, Hellenic Agricultural Organization “Demeter”, 11528 Athens, Greece; 2 Ekati Alchemy Lab SL, 08180 Moià, Spain; 3 Research Consultant, Ministry of Education, 11528 Athens, Greece

**Keywords:** genetic fidelity, microsatellites, simple sequence repeat, molecular markers, micropropagation, in vitro culture, indirect regeneration, somaclonal variation, hemp

## Abstract

Simple sequence repeat (SSR) markers were used to evaluate the genetic stability of the acclimatized micropropagated and regenerated plants of a high cannabidiol (H-CBD) and a high cannabigerol (H-CBG) variety of *Cannabis sativa* L. Shoot regeneration and proliferation were achieved by culturing calli in Murashige and Skoog basal medium (MS) supplemented with several concentrations of 6-benzyladenine (BA) or thidiazuron (TDZ). Calli derived mostly from stem explants, rather than leaves, cultured on MS supplemented with 2,4-Dichlorophenoxyacetic acid (2,4-D) or combination of kinetin (KIN) with 1-Naphthaleneacetic acid (NAA) or 2,4-D. Rooting of the regenerated plantlets accomplished on half-strength MS medium supplemented with indole-3-butyric acid (IBA). Previous studies performed have developed an efficient in vitro micropropagation protocol for mass production. Both in vitro methodologies can be employed in genetic breeding via molecular techniques. The genetic stability of micropropagated and regenerated plants was accomplished using twelve SSR primer pairs that produced reproducible and clear bands, ranging from 90 to 330 bp in size, and resulted in amplification of one or two alleles, corresponding to homozygous or heterozygous individuals. The SSR amplification products were monomorphic across all the micropropagated and regenerated plants and comparable to mother plants. The monomorphic banding pattern confirmed the genetic homogeneity of the in vitro cultured acclimatized and mother plants as no somaclonal variation was detected in clones for these specific SSRs. Our results evidently suggest that the developed culture protocols for in vitro multiplication is appropriate and applicable for clonal mass propagation of the *C. sativa* varieties and demonstrate the reliability of this in vitro propagation system.

## 1. Introduction

*Cannabis sativa* L., a multipurpose plant, i.e., with recreational, medicinal, and industrial usages, has been cultivated for at least 10,000 years, evolved along with man, and was declared as one of the oldest domestic plants in the history of mankind [1,2]. Hemp has spread worldwide and was recognized, at an early stage, as one of the most widely disseminated cultivated plants [3], provided that the conditions are suitable for its growth, becoming one of the most variable among cultivated plants [4]. Currently, Cannabis plant is used to produce more than 25,000 different products used for various purposes [5], with an emphasis on its medicinal use due to its pharmaceutical bioactive compounds.

Throughout the last decades, the detection of bioactive substances such as terpenoids, flavonoids and phytosterols [6], alkaloids and glycoproteins [7], and cannabinoids [8] found in its in-florescence’s glandular trichomes, has led to a significant increase in research on cannabis’ therapeutic potential and brought the species to the spotlight. A total of 565 Cannabis constituents have been isolated from *Cannabis sativa* so far [9] and approximately 150 compounds are considered as phytocannabinoids [10,11,12].

Aiming at increasing bioactive substances with medicinal properties in Cannabis, agricultural geneticists and breeders have detected and selected several cannabis varieties or strains that produce high amounts of cannabinoids such as cannabidiol (CBD) [13,14,15,16], cannabigerol (CBG) [15,16,17,18], and Δ^9^-tetrahydrocannabinol (Δ^9^-THC) [19,20,21]. This phenotypic selection would lead to the formation of varieties rich in specific phytocannabinoids [22], which should present phytochemical profile stability. These phenotypes with chemical profile consistency are introduced into the commercial production process. The propagation of such THC-, CBD- or CBG-enriched varieties can be achieved through in vitro culture techniques to provide, primarily, plant clonal propagation and, secondarily, a tool for plant improvement [23]. In vitro propagation enables rapid propagation of selected disease-free elite stock varieties in a relatively short period. Moreover, one of the main applications of in vitro culture is the preservation of genetic lines [24].

Although clonal material can be obtained by in vitro culture, this methodology can result in genetic variation as both medium composition and the use of plant growth regulators, such as auxins and cytokinins, induce somaclonal variation in propagated plants [24,25,26,27]. Moreover, clonal stability of regenerated plants is questioned by frequent transfers of cultures during micropropagation which might lead to genetic variation as well [28]. Eventually, in vitro conditions can result in developmental and physiological irregularities of the propagated plants [24]. The occurrence of somaclonal variation is an expected but unwanted drawback when the propagation of an elite germplasm is intended [29]. Genetic uniformity, i.e., clonal stability of micropropagated plants, is a prerequisite to maintain the advantages of desired elite genotypes and, therefore, for quality plant material production [26,28,29]. Thus, it is important to assess the genetic fidelity of the micropropagated and regenerated plants.

Various types of DNA-based molecular methods are involved in genetic polymorphism evaluation. Several molecular markers such as inter simple sequence repeat (ISSR), random amplified polymorphic DNA (RAPD), amplified fragment length polymorphism (AFLP), restriction fragment length polymorphisms (RFLP), and simple sequence repeats (SSR) have been used to detect the genetic fidelity and determine somaclonal variation in plants produced via micropropagation [28,30,31,32,33,34,35].

Of these molecular markers, SSR markers present overall distinguishing advantages as they are abundant in the genome, highly informative, codominant, multi-allele genetic markers, experimentally reproducible and transferable among related species, and they present wide applicability, easy interpretation in genotyping, easy automation, and PCR multiplexing ability [36,37,38,39,40]. Additionally, in contrast with other markers such as AFLPs, SNPs, and RFLPs, microsatellites do not require high-throughput technologies and computational resources for their development and analysis [39,40,41,42].

Over the last three decades, *Cannabis sativa* has been subjected to genetic analyses, i.e., characterization, marker-assisted selection, and individualization, via molecular markers based on RAPD [43,44], RFLP [45], AFLP [33], ISSR [29,46,47], and STR (short tandem repeat) [48,49,50,51,52,53,54,55,56,57,58,59]. The microsatellite markers for *C. sativa* that have been commonly used, as well as those newly developed [37], are very efficient in determining genetic diversity and can verify that plants obtained by in vitro culture are true-to-type to the mother plant from which they derived.

Previous studies performed by this scientific team [15,16] have successfully accomplished the development of efficient in vitro micropropagation protocols for mass production. Moreover, we have developed an in vitro plant regeneration process through callus formation, i.e., indirect organogenesis, which is presented in the present study. The main aim of this research was to assess the genetic stability of both micropropagated and regenerated plants based on the molecular analysis of SSR markers. Twelve selected highly polymorphic and discriminant SSRs were used to assess the genetic homogeneity of in vitro propagated and regenerated plants of two selected and chemically screened *Cannabis sativa* varieties with desirable characteristics—the first was rich in CBD (H-CBD) and the second in CBG (H-CBG).

## 2. Results

### 2.1. Plant Regeneration through Callus Formation

**Figure 1:**
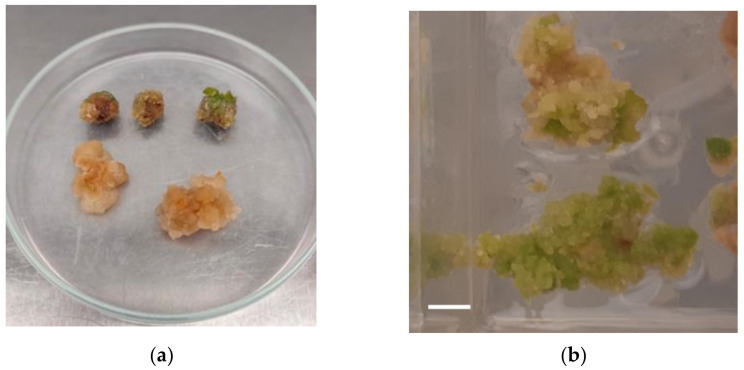
Callus induction: (**a**) Different appearance of calli depended on the callus induction medium. Petri dish diameter = 9 cm. (**b**) Callus induction in nodal segment explants of *Cannabis sativa* L. on MS medium containing 4.52 μΜ 2,4-D after 3 weeks of culture. Bar = 1 cm.

Calli (Figure 1) obtained from both tested types of explant, stems, and leaf sections of the H-CBD and H-CBG varieties. Stem explants presented the best efficiency of callus induction as all explants developed callus. In contrast, only slightly more than half of the explants originating from leaves developed a callus (54%). Plant growth regulators’ combination, concentration, and explant origin had significant effects on callus induction and plant regeneration. Callus initiation obtained after 2 weeks of culture when MS medium [60] was supplemented with several concentrations of 2,4-D (2.26 μM–9.04 μM) or a combination of KIN (1.16 μM–2.32 μM) with NAA (2.68 μM–5.37 μM) or 2,4-D (2.26 μM–4.52 μM) (Supplementary Figure S1). The appearance of calli as well as the frequency of callus induction, the time of callus initiation, and the rate of callus growth were different and depended on the callus induction medium. Callus color ranged from pale yellow to green and varied in character from friable or watery to compact (Figure 1).

**Figure 2:**
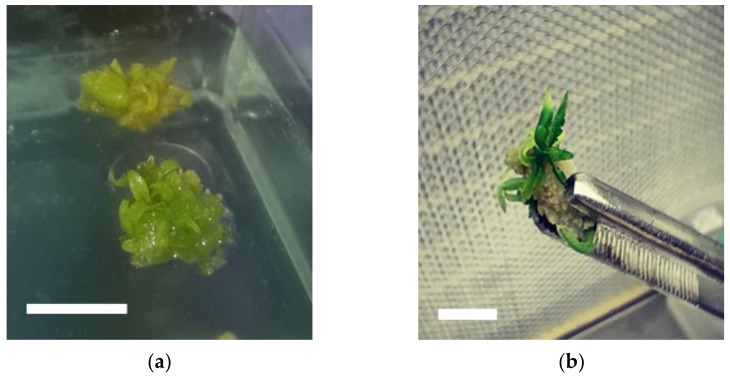
Shoot formation and plant regeneration from callus: (**a**) organogenesis in nodal segment explants of *Cannabis sativa* L. on MS medium containing 4.44 μΜ BA after 2 weeks of culture; (**b**) plant regeneration from callus formed in nodal segment explants on MS medium containing 4.44 μΜ BA after 3 weeks of culture. Bars = 1 cm.

Plantlets were regenerated mostly from nodular virescent callus derived from stem explants (Figure 2) after 3–4 weeks of culture on MS basal medium supplemented with several concentrations of BA (2.22 μM–8.88 μM) or TDZ (2.27 μM–9.08 μM) (Supplementary Figure S2). Sixty-two percent (174/279) of callii presented shoot formation (Supplementary Figures S2 and S3). Rooting of the regenerated plantlets accomplished after 2–3 weeks of culture on half-strength MS medium supplemented with indole-3-butyric acid (IBA) (Supplementary Figures S3 and S4). Overall, the percentage of rooted shoots regenerated from callii was relatively high: 73% (127/174). Best rooting results were obtained when half-strength MS medium was supplemented with 4 μM IBA.

Acclimatization of the plantlets was achieved in mini greenhouses after 3 weeks (Supplementary Figure S5). Only plantlets with well-developed roots were chosen (Supplementary Figure S3), and after being washed to remove agar (Supplementary Figure S4), they were placed in plastic pots, containing a sterile mixture of peat and pearlite (Supplementary Figure S5). Survival rate and rooting of regenerated plants as well as their acclimatization presented low percentages: ≤30% (37/127).

### 2.2. Genetic Data and Homogeneity Assessment

All SSR primers generated amplicons in all mother and in vitro cultured plants. The number of alleles and their range for each locus and *Cannabis sativa* variety are presented in Table 1.

**Figure 3:**
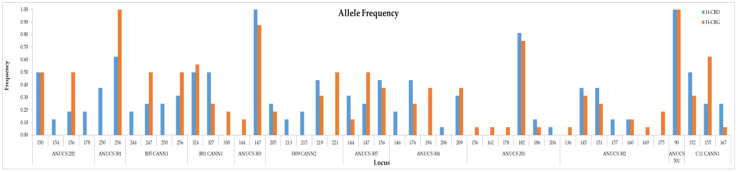
Allele frequencies for the two Cannabis varieties: the H-CBD and the H-CBG.

Overall, forty-five alleles were detected over all 12 STR loci for the 16 *Cannabis sativa* L. samples with an average of 3.75 ± 0.51 (mean ± standard error) alleles per locus. Alleles’ frequencies for the two Cannabis varieties, the H-CBD and the H-CBG, are presented in Figure 3. The mean of the different alleles per locus (Na) was calculated at 2.88 ± 0.27, while the effective alleles (Ne) was 2.30 ± 0.19 (Table 2). The mean observed (Ho) and the mean expected (He) heterozygosity were calculated at 0.45 ± 0.07 and 0.48 ± 0.05, respectivelly, while the fixation index *F* (inbreeding coefficient) was 0.06 ± 0.10. Shannon’s information index (I) was assessed at 0.82 ± 0.09. The percentage of polymorphic loci (PPL) in both varieties was estimated at 88.83%, and the *F_ST_* index was 0.07 ± 0.02. Nei genetic distance (Nei D) and Nei genetic identity (Nei I) was calculated at 0.145 and 0.865, respectively. The lowest polymorphism information content (PIC) values, if we exclude the zero values of ANUCS 303 and ANUCS 501, was observed in ANUCS 201 (0.294) and ANUCS 301 (0.359). The most informative marker was B05 CANN1 (PIC = 0.694) followed by the H09 CANN2 (PIC = 0.645). The polymorphism information content (PIC) values, as well as the observed heterozygosity (Ho) and the expected heterozygosity (He) for every marker, are presented in Table 2.

Concerning the H-CBD variety, 35 alleles were identified, of which 11 were unique, with an average of 2.92 ± 0.34 alleles per locus. The Ne was calculated at 2.44 ± 0.27. Ho and He were estimated at 0.48 ± 0.09 and 0.50 ± 0.08, respectively, while the inbreeding coefficient F was 0.05 ± 0.09. Shannon’s information index (I) was assessed at 0.87 ± 0.14. The amplification products ranged in size from 90 bp in ANUCS 501 to 327 bp in B01 CANN1. Two loci, ANUCS 303 and ANUCS 501, generated only one peak of 147 bp and 90 bp in size, respectively, while the rest of the primers led to two (ANUCS 301 and B01 CANN1) or more alleles per locus, with the highest number (4 alleles) being observed at ANUCS 202, B05 CANN1, H09 CANN2, ANUCS 304, and ANUCS 302 (Table 1). The lowest polymorphism information content (PIC) values, if we exclude the zero values of ANUCS 301 and ANUCS 501, was observed in ANUCS 303 (0.195). The most informative marker was ANUCS 302 (PIC = 0.748) followed by the ANUCS 304 (PIC = 0.582). The polymorphism information content (PIC) values, as well as the observed heterozygosity (Ho) and the expected heterozygosity (He) for every marker, are presented in Table 3.

**Figure 4:**
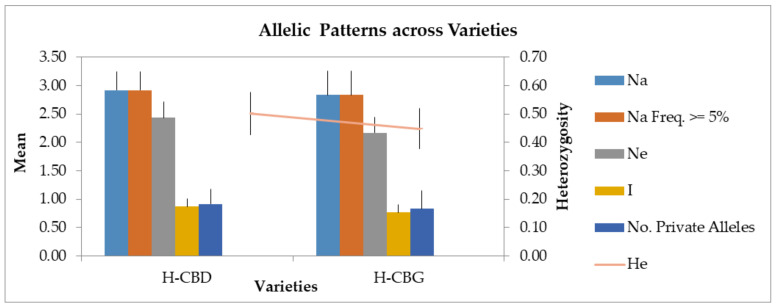
Allelic patterns across the H-CBD and the H-CBG Cannabis varieties (Na Freq ≥ 5% = Number of different alleles with a frequency ≥ 5%).

Overall, the most informative marker was ANUCS 302 (PIC = 0.717) followed by the H09 CANN2 (PIC = 0.693) and ANUCS 304 (PIC = 0.669). The lowest polymorphism information content values, if we exclude the zero value of ANUCS 501, was observed in ANUCS 303 (0.110) and ANUCS 301 (0.258). Moreover, allelic patterns across two Cannabis varieties, the H-CBD and the H-CBG, are presented in Figure 4. More information about genetic characteristics of mother plants of PCR amplicons for used STR loci and mother plant material of the two Cannabis sativa L. varieties, the H-CBD and the H-CBG variety, are presented in Supplementary Tables S1–S3.

**Figure 5:**
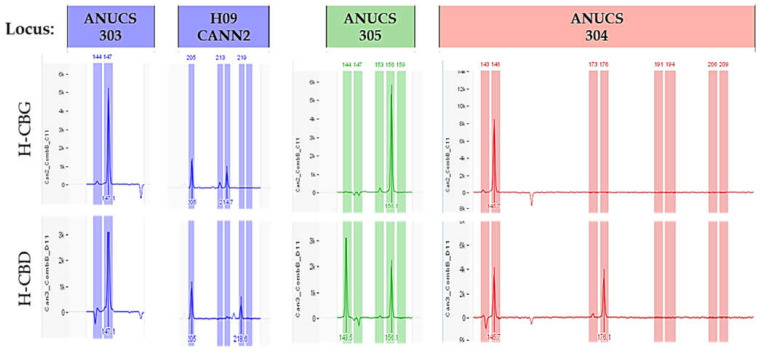
Depending on the genotype of each variety and the genetic locus, the plants showed homozygosity (H-CBG plant sample, loci: ANUCS 303, ANUCS 305, and ANUCS 304; H-CBD plant sample, locus ANUCS 303) or heterozygosity (H-CBG plant sample, locus: H09 CANN2; H-CBD plant sample, loci: H09 CANN2, ANUCS 305, and ANUCS 304).

Concerning the H-CBG variety, 34 alleles were identified, of which 10 were unique, with an average of 2.83 ± 0.42 alleles per locus. Ne was calculated at 2.17 ± 0.28. Ho and He were estimated at 0.42 ± 0.11 and 0.45 ± 0.07 (Table 3), respectively, with the inbreeding coefficient F at 0.07 ± 0.18. Shannon’s information index (I) was assessed at 0.77 ± 0.13. The amplification products ranged in size from 90 bp in ANUCS 501 to 330 bp in B01 CANN1. Two loci, ANUCS 301 and ANUCS 501, generated only one peak of 256 bp and 90 bp in size, respectively, while the rest of the primers led to two (ANUCS 202, B05 CANN1 and ANUCS 303) or more alleles per locus, and the highest number (6 alleles) was observed in ANUCS 302 (Table 1). Depending on the genotype of each variety and the genetic locus, donor and their cloned plants showed homozygosity or heterozygosity (Figure 5). 

**Figure 6:**
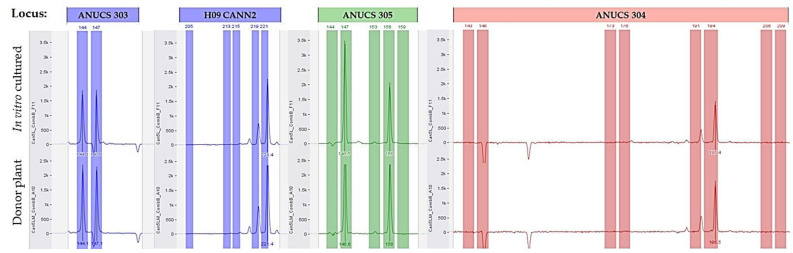
The simple sequence repeat amplification pattern obtained for mother plant (donor) and in vitro cultured micropropagated plant, using loci: ANUCS 303, H09 CANN2, ANUCS 305, and ANUCS 304.

**Figure 7:**
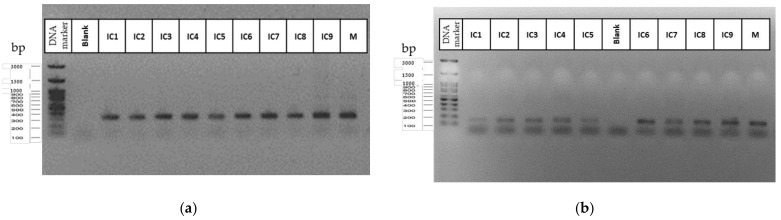
SSR amplification pattern obtained for the mother plant (M) and the in vitro cultured plants (I1–I9) for the SSR primers B01-CANN1 (**a**) and ANUCS 304 (**b**). The DNA marker is a 100 bp DNA ladder (100 bp–3000 bp) (FastGene 100 bp DNA Marker, Nippon Genetics Europe GmbH, Düren, Germany).

Micropropagated and regenerated plants showed no differences as compared with the mother plant in the SSR analysis. After 4 weeks of culture under environmental conditions, no SSR polymorphism was observed between donor genotypes and their plantlets. All primers presented standard allele patterns, regardless of the two in vitro culture methodologies. The genotyped in vitro cultured samples generated the same SSR profile to the original mother plant, i.e., the same alleles for all loci tested (Figure 6). Based on PCR assays for the 12 SSR primers used, no somaclonal variation was detected between the donor genotype and the in vitro cultured plants for each variety (Figure 7). Moreover, the general visual morphology of the acclimatized plants was also similar to mother plants.

## 3. Discussion

### 3.1. Plant Regeneration through Callus Formation

Callus induction and establishment, as well as their subculture and propagation, were straightforward. The use of MS supplemented with 2,4-D alone and also MS supplemented with KIN in combination with NAA or 2,4-D, proved to be efficient as callus induction treatment. The use of 2,4-D was found to give rise to callus, which was strongly organogenetic, something that was observed by Mandolino and Ranalli [61] in their own experiments. The results of the present study are in accordance with the findings of Ślusarkiewicz-Jarzina et al. [62] and Thacker et al. [63], although Ślusarkiewicz-Jarzina et al. [62] reported that DICAMBA produced the greatest amount of callus. Moreover, according to Feeney and Punja [64], the combination of KIN and 2,4-D created the optimal mixture of growth regulators which produced the greatest callus. On the contrary, our results were, to some extent, different from those of Lata et al. [65] who achieved the highest average callusing percentage using NAA. Furthermore, they found that the interaction of other auxins with TDZ was less effective. Similarly, Monthony et al. [66] and Wielgus et al. [67] both used NAA, but the former in combination with TDZ and the latter in combination with KIN, as callus induction medium. As stated by Wielgus et al. [67], the highest efficiency of morphogenic callus induction was observed from stem explants; this is in agreement to the present study.

Shoot regeneration and proliferation were achieved in MS supplemented with TDZ or BA in calli derived mostly from stem explants. In accordance with our results, Mandolino and Ranalli [61] promoted shoot formation using BA or 2,4-D in strongly organogenetic callus emerged on MS supplemented with 2,4-D. Contrary to Lata et al. [65], they did not obtain shoot formation from leaf-deriving callus. On the other hand, Lata et al., [65] using only leaf-deriving callus, found that shoot regeneration and proliferation were better in MS with TDZ, which is in accordance with our results, although they reported that media containing BAP or KN presented inferior results. Monthony et al. [66] repeating the treatments proposed by Lata et al. [65] but using different Cannabis genotypes, did not succeed in regeneration, although they have successfully induced callus. Wielgus et al. [67], using BAP in combination with NAA, succeeded in obtaining the highest percentage of plant regeneration from callus obtained from stem explant sources of *Cannabis sativa* L., too. On the contrary, Fisse et al. [68] assessed organogenesis, reporting that Cannabis calluses readily produced roots but were unreceptive to shoot formation. Moreover, Feeney and Punja [64] also failed to regenerate hemp plantlets from calli. However, the different genotypes, explant origin, and medium composition as well as the type, the concentration, and the combination of plant growth regulators, had important effects not only on callus induction but also on plant regeneration [61,62,64,65,66,67].

Root initiation of the well-developed in vitro regenerated plants was significantly influenced by supplementing the half-strength MS medium with IBA. Our results are in agreement with those of Lata et al. [65] who reported that the presence of IBA resulted in a significantly higher rooting percentage in the regenerated shoots. The presence of IBA was found to significantly promote rooting in *C. sativa* L. [30,69]. Numerous studies have also reported this promoting effect of IBA on in vitro rooting performance in several medicinal plants [70,71,72,73,74,75,76]. Conversely, instead of IBA, Ślusarkiewicz-Jarzina et al. [62] used a combination of IAA and NAA, and Wielgus et al. [67] used only IAA for root formation.

### 3.2. Genetic Data and Homogeneity Assessment

Allele peaks were observed in the size range as reported in the literature [48,52,54,57,58]. These results were in accordance with those of Gilmore and Peakall [49], Howard et al. [57], and Gilmore et al. [58], but somewhat different to those of Köhnemann et al. [54]. The most diverse locus was ANUCS 302. ANUCS 201 and H09 CANN2 presented a high allele number, too, findings that were comparable to the results of Gilmore and Peakall [49] and Gilmore et al. [58].

Using this specific SSR system, it is possible to analyze and collect different Cannabis DNA profiles, allowing discrimination among accessions, even individuals [49], and possibly identifying geographical differences [54]. Our results presented, to some extent, allele frequency estimations of the two hemp varieties, although they do not preview an extensive population study due to the limited number of varieties and samples included in the analysis. The two varieties that were used as the experimental material are under development in a breeding project. Having in mind in vitro culture plants with maximum possible content in cannabinoids, i.e., CBD and CBG but not THC, we screened a group of plants with only desirable traits and eventually only elite, based on chemical profile, female plants were chosen, which are engaged in our experiments. All the plant material were from the private collection of the company financing the project. Therefore, the genetic base of the in vitro cultured plants was rather narrow which is why only forty-five alleles were detected using the twelve set of SSR primers. Though, the genetic diversity between the two *C. sativa* varieties is very low due to a low *F_ST_* value [77]. This conclusion is confirmed by both the low value of Nei D and the high value of Nei I indices [78,79], suggesting the genetic similarity of the two varieties and indicating that both varieties belong to the same population. Furthermore, as the *F* value is close to zero, it could be assumed that the plants of both varieties come from populations that are under random mating [80]. 

A critical factor in in vitro plant micropropagation and regeneration is to evaluate their genetic fidelity. During the in vitro culture conditions for extended periods, the plant material might present somaclonal variation [81], particularly in plantlets regenerated from callus [82]. This phenomenon raises greater concern in large-scale production of economically important crop plants or in genetic resources conservation [9,29,81,83,84,85,86,87,88]. Somaclonal variation may limit the effectiveness of any micropropagation program where it is important to produce true-to-type plant material [85,88,89,90]. Thus, it is crucial to assess the genetic uniformity of in vitro propagated plantlets. This can be achieved by DNA analysis, without the need of DNA sequence information, in order to confirm that the plantlets are genetically identical to the donor plant [91]. Among the plethora of PCR-based marker approaches to assess the genetic stability of in vitro cultured plants, many studies suggested that SSRs are capable of identifying whether two single plants are bred from the same mother plant [54]. This occurs because *Cannabis sativa* is often propagated clonally, mainly to protect the genetic identity of the cultured varieties and, moreover, for practical reasons [51]. As a result, both mother plant and subsequent daughter clones have identical DNA profiles that are easy comparable via SSR marker analysis [48]. The findings of several studies clearly demonstrated this potential [49,55,58].

All the SSR profiles of the acclimatized micropropagated and regenerated plants were identical, i.e., true-to-type to the donor plant, indicating their genetic homogeneity. However, it should be emphasized that by using various molecular markers, including SSRs, we cannot effectively detect all clonal variability originated by all possible random mutations that occurred in Cannabis genome due to its size. The estimated haploid genome sizes of a male and a female plant are approximately 843 Mb and 818 Mb, respectively [92], although this depends, among others, on the variety or the assessment method [93,94]. Nevertheless, similar studies on the genetic stability of regenerated plants of several species using molecular markers revealed genetic fidelity. Simple sequence repeats markers were employed to assess the genetic uniformity of the micropropagated plants of *Helianthus verticillatus* Small, and their genetic stability was confirmed between the regenerants and their respective donor plants [95]. Kakimzhanova et al. [96] reported that simple sequence repeat analysis confirmed the reliability (genetic homogeneity) of their protocol for efficient large-scale micropropagation of *Malus sieversii*. In another species of *Malus*, *Malus niedzwetzkyana*, Nurtaza et al. [97] using simple sequence repeat primers, detected no somaclonal variation between the mother plant and the micropropagated clones of this endangered species. Asadi-Aghbolaghi et al. [98] analyzing the genetic stability of regenerated *Stipagrostis pennata* plants using SSR markers, detected no somaclonal variation in regenerated plants from somatic embryos of *S. pennata*. Bandupriya et al. [99] using SSR markers, stated that there was no somaclonal variation or genetic instability in plantlets that were regenerated from *Cocos nucifera* L. ovary explants. Wanmei et al. [100], evaluating the genetic fidelity of grape-regenerated plantlets, detected no polymorphism, also indicating the genetic fidelity of regenerated plants. Moreover, Pandey et al. [101] assessing the genetic fidelity in sugarcane (*Saccharum officinarum*), found that SSR patterns of the regenerated plantlets through direct organogenesis, were identical to those of the mother plant, indicating that direct adventitious organogenesis did not induce somaclonal variation. Rai et al. [102], who evaluated the genetic fidelity of *Psidium guajava* L. (guava) plants developed from in vitro somatic embryogenesis using six SSR primer pairs, reported that the amplification products were monomorphic across all the regenerated plants. In addition, Wanmei et al. [103], using SSR markers, found that all banding profiles from regenerated plantlets of *Malus hupehensis* var. *pinyiensis* were monomorphic and identical to those of the donor plant, showing genetic uniformity of the in vitro cultured plantlets. Tiwari et al. [104], assessing the genetic stability of in vitro conserved potato microtubers by SSR, found that DNA analyses revealed 100% similarity among mother plant and its derivatives by SSR markers, indicating a true-to-type progeny. Castillo et al. [105] examined the genetic stability of cryopreserved shoot tips of *Rubus grabowskii* germplasm; no SSR polymorphisms were observed between cryopreserved shoots and the corresponding mother plants regardless of subculture. In *Cannabis sativa*, Chandra et al. [106], using a set of seven amplified SSR markers, reported that all the tested clones, derived from several mother plants of different varieties, were easily identified. Moreover, in two studies, Lata et al. [29,47], having a different approach, used ISSR to evaluate the genetic stability of the micropropagated plants and found that all the profiles from micropropagated plants were monomorphic and comparable to mother plants, confirming the genetic stability. 

The results of the present study show that the micropropagation and regeneration protocol can be a useful method for culturing genetically uniform plants. Furthermore, the visual observations of the micropropagated and regenerated plants did not show any variations compared with mother plants.

## 4. Materials and Methods

### 4.1. Plant Material

Two varieties, a high-cannabidiol (H-CBD) and a high-cannabigerol (H-CBG) of *C. sativa* L., were included in the present study. Ekati Alchemy Lab SL (Barcelona, Spain) kindly provided these varieties. The selected elite (based on chemical profile) female donor (mother) plants and their cloned acclimatized plants, originating from in vitro micropropagation, were grown in a greenhouse located at the Institute of Mediterranean and Forest Ecosystems Hellenic Agricultural Organization “Demeter” in Athens, Greece. All the details regarding the in vitro micropropagated plants are described extensively in the context of a previous research study [15]. Eight mother plants from both the H-CBD and H-CBG variety and nine micropropagated plants per donor plant were used in the experiments. All plants were kept indoors, at the vegetative stage with a 16 h/8 h (light/dark) photoperiod, under controlled environmental conditions at 27 °C ± 2 °C, having approximately 500 μmol m^−2^ s^−1^ photosynthetic photon flux density at culture level.

### 4.2. Plant Regeneration through Callus Formation

Stems ~1.0 cm long and leaf sections ~1.0 cm^2^ from cannabis plants were grown in a growth chamber at 23 ± 1 °C under a 16 h/8 h (light/dark) photoperiod. They were surface-sterilized through successive immersion in 1.0% NaOCl (*v/v*) (10% NaOCl, Fluka, Buchs, Switzerland) supplemented with 0.05% (*v/v*) Tween-20 (Fisher Bioreagents, Pittsburgh, PA, USA), with continuous stirring for 12 min, followed by immersion in 70% ethanol (*v/v*) for 45 s. Each immersion was followed by three rinses with sterile deionized water that lasted three minutes. The disinfection process took place under sterile conditions, as did all the downstream handlings. 

Stems and leaf sections were implanted in magenta vessels (77 mm × 77 mm × 77 mm) (Sigma Aldrich, Merck KGaA, Steinheim am Albuch, Germany). The used MS basal medium [60], was supplemented with various concentrations (0.54 μM–9.12 μM) of plant growth regulators, i.e., 2,4-Dichlorophenoxyacetic acid (2,4-D), 1-Naphthaleneacetic acid (NAA), kinetin (KIN), 6-benzyladenine (BA), and thidiazuron (TDZ) (Sigma Chemicals, Saint Louis, MO, USA or Duchefa Biochemie, Haarlem, The Netherlands), alone or in combination (data not shown). Growth regulators were added by filter sterilization after the media was autoclaved. Cultures were kept in darkness at 23 ± 1 °C for 3–4 weeks. After callus induction, calli were excised from the original explants and transferred on the same fresh medium. The new media were supplemented with various concentrations (0.44 μM–8.88 μM) of BA, TDZ and KIN, alone or in combination. The cultures were incubated in a growth chamber at 23 ± 1 °C, under a 16 h/8 h (light/dark) photoperiod and under LED grow lights of 50 μmol m^−2^ s^−1^ photosynthetic photon flux density at culture level until adventitious shoot formation occurred. For root formation, regenerated plantlets ~2 cm high were excised from calli and cultured on half-strength MS basal medium supplemented with IBA at various concentrations (1.0 μM–8.0 μM).

Plants with well-developed roots, were transplanted in 6.5 cm × 6.5 cm × 8.0 cm plastic pots containing a 3 peat: 1 pearlite (*v/v*) sterile mixture, after washing the roots to remove agar. The pots were placed in 48 cm × 33 cm × 20 cm mini greenhouses (Nortene, Ballée, France) with plastic covers in order to avoid water loss and maintain humidity. All the plantlets were kept under controlled environmental conditions at 27 ± 2b °C with a 16 h/8 h (light/dark) photoperiod, with an approximately 50 μmol m^−2^ s^−1^ photosynthetic photon flux density at culture level, supplied by LED grow lights. The acclimatized plantlets were transplanted to flowerpots and placed indoors, under controlled environmental conditions at 27 ± 2 °C, a 16 h/8 h (light/dark) photoperiod and under LED grow lights of 500 μmol m^−2^ s^−1^ photosynthetic photon flux density at culture level.

### 4.3. DNA Isolation and Quantification

Fresh leaf samples, approximately 100 mg each, were frozen in liquid nitrogen, ground into a fine powder using clean and sterile pestle and mortar, and then stored in 2.0-mL microcentrifuge tube. Total genomic DNA was extracted using the NucleoSpin Plant II kit (Macherey-Nagel, Düren, Germany) following the manufacturer’s instructions and finally resuspended in 50 μL elution buffer provided by the kit. Prior to storage in −20 °C, the purified total DNA of the samples was quantified, and its quality was verified by using the micro-volume UV-Vis spectrophotometer Q5000 UV-Vis (Quawell, San Jose, CA, USA).

### 4.4. Microsatellite Loci

Twelve selected highly polymorphic and discriminant microsatellite loci [48,49,51,55,58] were analyzed to assess the genetic homogeneity of in vitro cultured plants. The sequences and characteristics for 12 *Cannabis sativa* microsatellite loci are presented in Table 4.

### 4.5. PCR Reaction Mix and Amplification

The PCR reaction was performed in a total volume of 20.0 μL comprising 30 ng genomic DNA. The final concentrations of reaction mix components were 1xPCR buffer (10x) (Nippon Genetics, Tokyo, Japan), 4 mM of MgCl_2_ (50 mM) (Nippon Genetics, Tokyo, Japan), including the amount of MgCl_2_ contained in PCR buffer, 1U Fast Gene Taq DNA polymerase (5 U μL^−1^, Nippon Genetics, Tokyo, Japan), 250 μM each of dNTPs (10 mM) (Nippon Genetics, Tokyo, Japan), and 0.2 μM of each forward and reverse primer (Eurofins Genomics, Ebersberg, Germany). 

Amplifications were carried out in Bio Rad C1000 Touch PCR thermal cycler (Bio-Rad, Hercules, CA, USA), programmed at 95 °C for 10 min for initial denaturation, followed by 30 cycles of 30 s at 95 °C, 90 s at annealing temperature of 60 °C, 1 min at extension temperature of 72 °C, and a final step of extension of 30 min at 72 °C. Final holding temperature was 4 °C. All samples were analyzed twice. Every set of PCR reactions included one negative and one positive control.

### 4.6. Capillary Electrophoresis, Genotyping and Statistical Data

Fragment analysis, separation, and detection of PCR products, were performed on the 3730 Genetic Analyzer (Applied Biosystems, Thermo Fisher Scientific Co., Waltham, MA, USA). An aliquot (1 μL) of PCR product was added to 9 μL of cocktail, i.e., 8.5 μL Hi-Di Forma-mide^®^ and 0.5 μL LIZ^®^ 500 Size Standard (Applied Biosystems, Thermo Fisher Scientific Co., Waltham, MA, USA). Samples were then denatured for 3 min at 95 °C and immediately chilled on ice for 2 min and loaded on the 3730 Genetic Analyzer (Applied Biosystems, Thermo Fisher Scientific Co., Waltham, MA, USA) and run using the following conditions: oven at 63 °C; pre-run 15 kV, 180 s; injection 1.6 kV, 5 s; run 15 kV, 1600 s; capillary length 50 cm; polymer: POP-7™; and dye set G5. A customized bin set was designed, and an allelic ladder (generated from sequence data for each marker) was included with each injection to ensure accurate genotyping. Genotyping was performed using Geneious Prime v. 2022.1.1 software (Dotmatics, Boston, MA, USA). The analytical threshold was set at 150 relative fluorescence units (RFUs).

Several informative parameters such as the number of observed alleles (Na), effective number of alleles (Ne), Shannon’s information index (I), percentage of polymorphic loci (PPL), Nei genetic distance (Nei D), and Nei Genetic Identity (Nei I) were estimated using the GenAlEx package [107]. Moreover, the polymorphism information content (PIC) value for every marker in each variety as well as for all samples, as defined by Botstein et al., was determined [108] using Cervus 3.0.7 [109]. Hardy–Weinberg tests were not conducted due to small within-population sample sizes.

## 5. Conclusions

An in vitro regeneration protocol was developed for the two selected high CBD and CBG *Cannabis sativa* varieties. The regeneration method demonstrated rather low frequency of shoot formation and multiplication as well as survival rate of rooted plantlets during acclimatization. Along with the previously efficient in vitro micropropagation protocol that was developed and can be used in industrial cultivation for the large-scale production, both in vitro methodologies are able to be employed in genetic breeding via molecular techniques. Moreover, the SSR markers used to assess the genetic fidelity of in vitro cultured plants revealed that the banding pattern of PCR amplified products were monomorphic and comparable to mother plants. The results confirmed the genetic stability among clones and mother plants as no somaclonal variation was detected in clones for these specific SSRs. Our results clearly suggest that the culture protocols developed for in vitro multiplication is appropriate and applicable for clonal mass propagation of the *C. sativa* varieties. 

## Figures and Tables

**Table 1:** Characteristics of PCR amplicons for studied STR loci and plant material of *Cannabis sativa* L. varieties.

STR Locus ^1^	H-CBD		H-CBG		Overall	
	Number of Alleles	Range (bp ^2^)	Number of Alleles	Range (bp ^2^)	Number of Alleles	Range (bp ^2^)
ANUCS 202	4	150–178	2	150–156	4	150–178
ANUCS 301	2	250–256	1	256–256	2	250–256
B05 CANN1	4	244–256	2	247–256	4	244–256
B01 CANN1	2	324–327	3	324–330	3	324–330
ANUCS 303	1	147–147	2	144–147	2	144–147
H09 CANN2	4	205–219	3	205–221	5	205–221
ANUCS 305	3	144–156	3	144–156	3	144–156
ANUCS 304	4	146–209	3	176–209	5	146–209
ANUCS 201	3	182–204	5	156–186	6	156–204
ANUCS 302	4	145–160	6	136–175	7	136–175
ANUCS 501	1	90–90	1	90–90	1	90–90
C11 CANN1	3	152–167	3	152–167	3	152–167

^1^ Short tandem repeat, ^2^ bp = base pairs.

**Table 2:** Genetic informative parameters of *Cannabis sativa* L. varieties.

Variety		n	Na	Ne	I	Ho	He	F	Percentage of Polymorphic Loci	Nei D	Nei I	Fst
**H-CBD**	**Mean**	8	2.92	2.44	0.87	0.48	0.50	0.05	83.33%	0.145	0.865	0.075
	**SE**		0.34	0.27	0.14	0.09	0.08	0.09				
**H-CBG**	**Mean**	8	2.83	2.17	0.77	0.42	0.45	0.07	83.33%			
	**SE**		0.42	0.28	0.13	0.11	0.07	0.18				
**Overal**	**Mean**	8	2.88	2.30	0.82	0.45	0.48	0.06	83.33%			
	**SE**		0.27	0.19	0.09	0.07	0.05	0.10	0.00%			

n = Number of samples, Na = Number of Different Alleles, Ne = Number of Effective Alleles, I = Shannon’s Information Index, F = Fixation Index, Ho = Observed Heterozygosity, He = Expected Heterozygosity, Nei D = Nei’s Genetic Distance, Nei I = Nei’s Genetic Identity, F_ST_ = inbreeding coefficient.

**Table 3:** Characteristics of studied STR locus and plant material of *Cannabis sativa* L. varieties.

Population	H-CBD			H-CBG			Overall		
Locus	Ho ^1^	He ^2^	PIC ^3^	Ho ^1^	He ^2^	PIC	Ho ^1^	He ^2^	PIC
ANUCS 202	0.500	0.664	0.616	0.000	0.500	0.375	0.250	0.582	0.551
ANUCS 301	0.250	0.469	0.359	0.000	0.000	0.000	0.125	0.234	0.258
B05 CANN1	1.000	0.742	0.694	1.000	0.500	0.375	1.000	0.621	0.608
B01 CANN1	0.500	0.500	0.375	0.875	0.586	0.520	0.688	0.543	0.482
ANUCS 303	0.000	0.000	0.000	0.250	0.219	0.195	0.125	0.109	0.110
H09 CANN2	0.500	0.695	0.645	0.375	0.617	0.544	0.438	0.656	0.693
ANUCS 305	0.625	0.648	0.575	0.750	0.594	0.511	0.688	0.621	0.571
ANUCS 304	0.500	0.672	0.612	0.250	0.656	0.582	0.375	0.664	0.669
ANUCS 201	0.375	0.320	0.294	0.375	0.422	0.404	0.375	0.371	0.361
ANUCS 302	1.000	0.688	0.630	1.000	0.781	0.748	1.000	0.734	0.717
ANUCS 501	0.000	0.000	0.000	0.000	0.000	0.000	0.000	0.000	0.000
C11 CANN1	0.500	0.625	0.555	0.125	0.508	0.428	0.313	0.566	0.539

^1^ Ho: observed heterozygosity, ^2^ He: expected heterozygosity, ^3^ PIC: polymorphism information content.

**Table 4:** Primer sequences and characteristics for 12 *Cannabis sativa* microsatellite loci.

Locus	Repeat Motif	Primer Sequences 5′→3′		Expected Allele Size Range (bp *)
		Forward	Reverse	
ANUCS 201	(GA)	GGTTCAATGGAGATTCTCGT	CCACTAAACCAAAAGTACTCTTC	209–265
H09-CANN2	(GA)	CGTACAGTGATCGTAGTTGAG	ACACATACAGAGAGAGCCC	104–113
ANUCS 303	(GTG)	TAATCAACAATGACAATGGC	GATTAAGGTCCTCGACGATA	314–349
ANUCS 305	(TGG)	AAAGTTGGTCTGAGAAGCAAT	CCTAGGAACTTTCGACAACA	140–200
ANUCS 301	(TTA)	ATATGGTTGAAATCCATTGC	TAACAAAGTTTCGTGAGGGT	141–162
ANUCS 304	(TCT)_x_TCA(TCT)_y_	TCTTCACTCACCTCCTCTCT	TCTTTAAGCGGGACTCGT	235–245
ANUCS 501	(TTGTG)	AGCAATAATGGAGTGAGTGAAC	AGAGATCAAGAAATTGAGATTCC	285–297
ANUCS 302	(ACA)_x_(ACA)_y_(ACA)_z_	AACATAAACACCAACAACTGC	ATGGTTGATGTTTTGATGGT	163–189
C11-CANN1	(TGA)_x_(TGG)_y_	GTGGTGGTGATGATGATAATGG	TGAATTGGTTACGATGGCG	120–242
ANUCS 202	(GA)	AGGACCAATTTTGAATATGC	AGAGAGGGAAGGGCTAACTA	140–230
B01-CANN1	(GAA)_x_A(GAA)_y_	TGGAGTCAAATGAAAGGGAAC	CCATAGCATTATCCCACTCAAG	140–230
B05-CANN1	(TTG)	TTGATGGTGGTGAAACGGC	CCCCAATCTCAATCTCAACCC	120–267

* bp = base pair.

## Data Availability

Data not available.

## Supplementary Materials

The following supporting information can be downloaded at: https://www.mdpi.com/article/10.3390/plants11192569/s1, Figure S1. Callus induction and subculturing: (a) stem and leaf explants placed in callus induction medium (1× MS basal medium supplemented with 2.25 μΜ Kin and 4.5 μΜ 2,4 D). Petri dish diameter = 9 cm and (b) Callii subculturing in magenta vessel on MS medium containing 4.52 μΜ 2,4-D after 3 weeks of culture. Magenta vessel bottom dimensions = 7 cm × 7 cm; Figure S2. Shoot formation and plant regeneration from callus in stem explants: (a) and (b) shoot organogenesis in stem segment explants of *Cannabis sativa* L. on MS medium containing 4.44 μΜ BA after 2 and 3 weeks of culture, respectively, and (c) multiple shoots formation on MS basal medium supplemented with 4.54 μM TDZ. Petri dish diameter = 9 cm; Figure S3. Rooting of shoots regenerated from callii: (a) root induction. Test tube diameter = 25 mm. and (b–d) root elongation on MS basal medium supplemented with several plant growth regulators. Test tube diameter = 25 mm. Magenta vessel bottom dimensions = 7 cm × 7 cm; Figure S4. Rooted shoots regenerated from callii ready for acclimatization; Figure S5. Survived plantlet regenerated from callii placed in plastic pot containing a sterile mixture of peat and pearlite after three weeks of acclimatization; Table S1. Several genetic characteristics of PCR amplicons for used STR loci and mother plant material of the two *Cannabis sativa* L. varieties, i.e., the H-CBD and the H-CBG variety; Table S2. Several genetic characteristics of PCR amplicons for used STR loci and mother plant material of the total *Cannabis sativa* L. samples; Table S3. Mean genetic characteristics of PCR amplicons for used STR loci and mother plant material of the two *Cannabis sativa* L. varieties, i.e., the H-CBD and the H-CBG variety, as well as for total donor plants.
