# Supplementary material for: Genetic Evaluation of In Vitro Micropropagated and Regenerated Plants of Cannabis sativa L. Using SSR Molecular Markers
Source: Plants (Basel). 2022 Sep 29;11(19):2569. doi: 10.3390/plants11192569 (PMC9573407; doi:10.3390/plants11192569)
Supplement: Supplementary file 1 [file plants-11-02569-s001.zip › plants-1828544-supplementary.pdf]

plants

Article

Genetic Evaluation of In Vitro Micropropagated and Regenerated Plants of *Cannabis sativa* L. Using SSR Molecular Markers

Kostas Ioannidis[1,*], Ioanna Tomprou[2], Vangelis Mitsis[2], Polyxeni Koropouli[3]

1 Laboratory of Sylviculture, Forest Genetics and Biotechnology, Institute of Mediterranean and Forest Ecosystems, Hellenic Agricultural Organization "Demeter", Ilissia, 11528 Athens, Greece
2 Ekati Alchemy Lab SL, Barcelona, Spain
3 Research consultant
* Correspondence: ioko@fria.gr; Tel +30-210-7783-750 (K.I.)

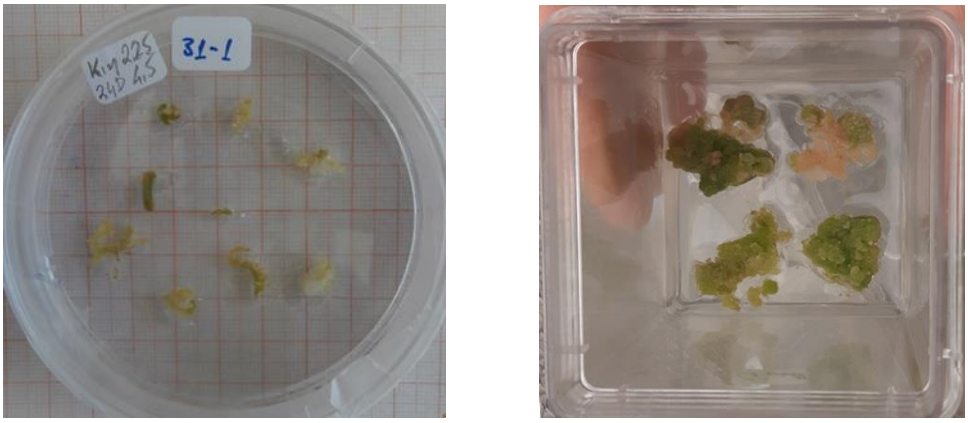

(**a**) (**b**)

Figure S1. Callus induction and subculturing: (a) stem and leaf explants placed in callus induction medium (1x MS basal medium supplemented with 2.25 μM Kin and 4.5 μM 2,4 D). Petri dish diameter = 9 cm. (b) Callii subculturing in magenta vessel on MS medium containing 4.52 μM 2,4-D after 3 weeks of culture. Magenta vessel bottom dimensions = 7 cm X 7 cm.

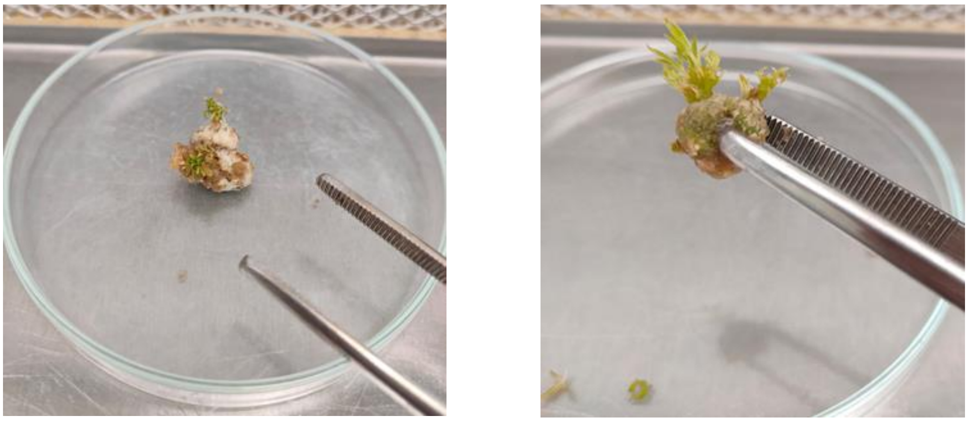

(**a**) (**b**)

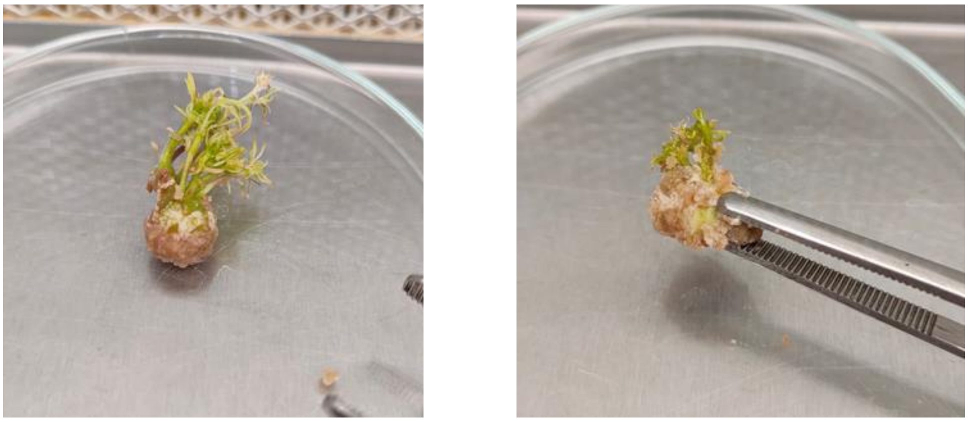

(**c**) (**d**)

Figure S2. Shoot formation and plant regeneration from callus in stem explants: (a) and (b) shoot organogenesis in stem segment explants of *Cannabis sativa* L. on MS medium containing 4.44 µM BA after 2 and 3 weeks of culture respectively; (c) Multiple shoots formation on MS basal medium supplemented with 4.54 µM TDZ. Petri dish diameter = 9 cm.

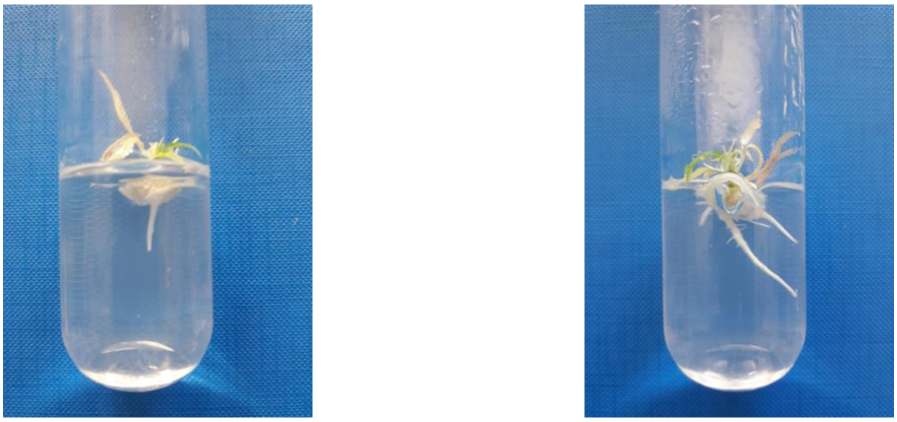

(a) **(b)**

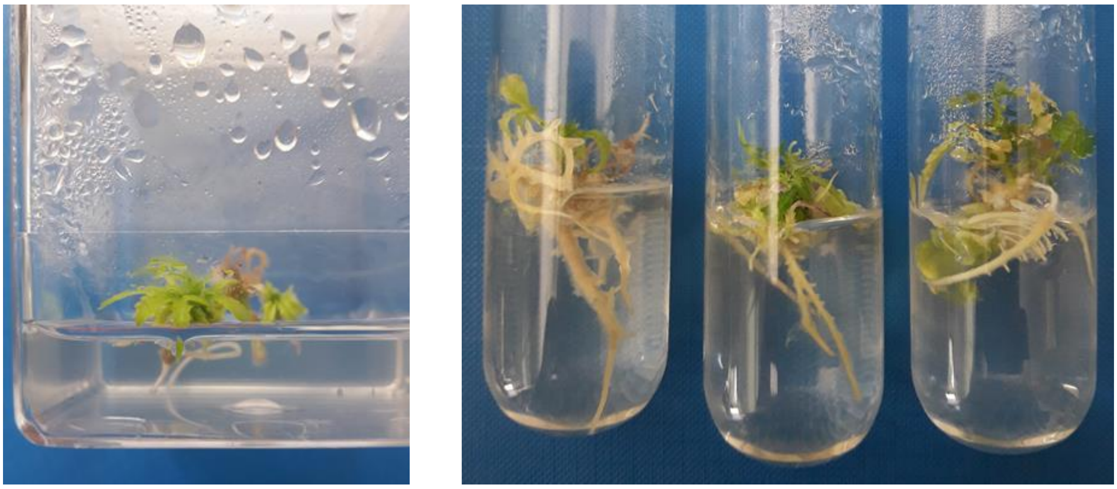

(**c**) (**d**)

Figure S3. Rooting of shoots regenerated from callii: (a) root induction. Test tube diameter = 25 mm.; (b-d) root elongation on MS basal medium supplemented with several plant growth regulators. Test tube diameter = 25 mm. Magenta vessel bottom dimensions = 7 cm X 7 cm.

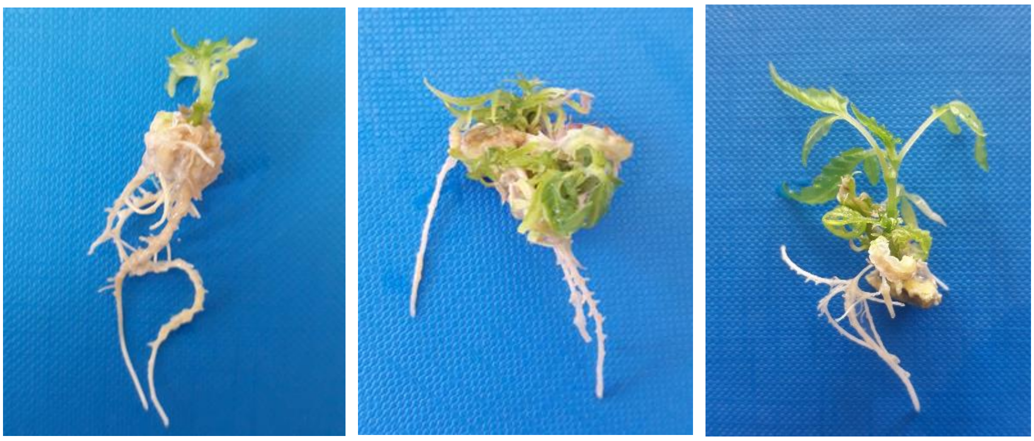

Figure S4. Rooted shoots regenerated from callii ready for acclimatization.

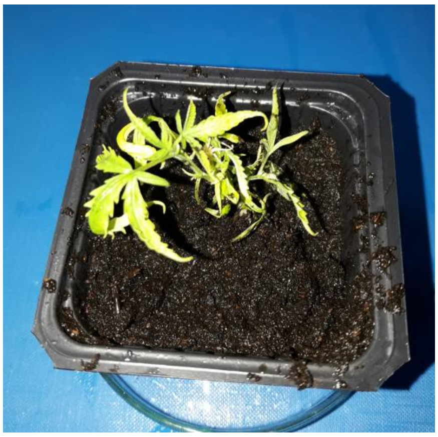

Figure S5. Survived plantlet regenerated from callii placed in plastic pot, containing a sterile mixture of peat and pearlite after three weeks of acclimatization.

Table S1. Several genetic characteristics of PCR amplicons for used STR loci and mother plant material of the two *Cannabis sativa* L. varieties, i.e. the H-CBD and the H-CBG variety.

Variety	Locus	n	Na	Ne	I	Ho	He	uHe	F	PIC
H-CBD	ANUCS 202	8	4	2.977	1.234	0.500	0.664	0.708	0.247	0.616
	ANUCS 301	8	2	1.882	0.662	0.250	0.469	0.500	0.467	0.359
	B05 CANN1	8	4	3.879	1.371	1.000	0.742	0.792	-0.347	0.694
	B01 CANN1	8	2	2.000	0.693	0.500	0.500	0.533	0.000	0.375
	ANUCS 303	8	1	1.000	0.000	0.000	0.000	0.000	-	0.000
	H09 CANN2	8	4	3.282	1.282	0.500	0.695	0.742	0.281	0.645
	ANUCS 305	8	3	2.844	1.072	0.625	0.648	0.692	0.036	0.575
	ANUCS 304	8	4	3.048	1.212	0.500	0.672	0.717	0.256	0.612
	ANUCS 201	8	3	1.471	0.602	0.375	0.320	0.342	-0.171	0.294
	ANUCS 302	8	4	3.200	1.255	1.000	0.688	0.733	-0.455	0.630
	ANUCS 501	8	1	1.000	0.000	0.000	0.000	0.000	-	0.000
	C11 CANN1	8	3	2.667	1.040	0.500	0.625	0.667	0.200	0.555
H-CBG	ANUCS 202	8	2	2.000	0.693	0.000	0.500	0.533	1.000	0.375
	ANUCS 301	8	1	1.000	0.000	0.000	0.000	0.000	-	0.000

B05 CANN1	8	2	2.000	0.693	1.000	0.500	0.533	-1.000	0.375
B01 CANN1	8	3	2.415	0.984	0.875	0.586	0.625	-0.493	0.520
ANUCS 303	8	2	1.280	0.377	0.250	0.219	0.233	-0.143	0.195
H09 CANN2	8	3	2.612	1.024	0.375	0.617	0.658	0.392	0.544
ANUCS 305	8	3	2.462	0.974	0.750	0.594	0.633	-0.263	0.511
ANUCS 304	8	3	2.909	1.082	0.250	0.656	0.700	0.619	0.582
ANUCS 201	8	5	1.730	0.909	0.375	0.422	0.450	0.111	0.404
ANUCS 302	8	6	4.571	1.630	1.000	0.781	0.833	-0.280	0.748
ANUCS 501	8	1	1.000	0.000	0.000	0.000	0.000	-	0.000
C11 CANN1	8	3	2.032	0.831	0.125	0.508	0.542	0.754	0.428

n = Number of samples, Na = Number of Different Alleles, Ne = Number of Effective Alleles, I = Shannon's Information Index, Ho = Observed Heterozygosity, He = Expected Heterozygosity, uHe = Unbiased Expected Heterozygosity, F = Fixation Index, PIC = polymorphism information content.

Table S2. Several genetic characteristics of PCR amplicons for used STR loci and mother plant material of the total *Cannabis sativa* L. samples.

Locus	n	Ht	Mean He	Mean Ho	Fis	Fit	Fst	PIC
ANUCS 202	16	0.619	0.582	0.250	0.570	0.596	0.060	0.551
ANUCS 301	16	0.305	0.234	0.125	0.467	0.590	0.231	0.258
B05 CANN1	16	0.670	0.621	1.000	-0.610	-0.493	0.073	0.608
B01 CANN1	16	0.568	0.543	0.688	-0.266	-0.210	0.045	0.482
ANUCS 303	16	0.117	0.109	0.125	-0.143	-0.067	0.067	0.110
H09 CANN2	16	0.736	0.656	0.438	0.333	0.406	0.109	0.693
ANUCS 305	16	0.646	0.621	0.688	-0.107	-0.063	0.039	0.571
ANUCS 304	16	0.719	0.664	0.375	0.435	0.478	0.076	0.669
ANUCS 201	16	0.377	0.371	0.375	-0.011	0.005	0.016	0.361
ANUCS 302	16	0.754	0.734	1.000	-0.362	-0.326	0.026	0.717
ANUCS 501	16	0.000	0.000	0.000	-	-	-	0.000
C11 CANN1	16	0.619	0.566	0.313	0.448	0.495	0.085	0.539

n = Number of samples, Ht = Total Expected Heterozygosity, Mean He = Mean Expected Heterozygosity, Mean Ho = Mean Observed Heterozygosity, Fis = inbreeding coefficient within individuals, Fit = inbreeding coefficient within individuals relative to the total, Fst = inbreeding coefficient, PIC = polymorphism information content.

Table S3. Mean genetic characteristics of PCR amplicons for used STR loci and mother plant material of the two *Cannabis sativa* L. varieties, i.e. the H-CBD and the H-CBG variety, as well as for total donor plants.

Variety	Statistic	n	Na	Ne	I	Ho	He	uHe	F	Percentage of Polymorphic Loci	Nei D	Nei I	Fst
H-CBD	Mean	8	2.92	2.44	0.87	0.48	0.50	0.54	0.05	83.33%	0.145	0.865	0.075

	SE		0.34	0.27	0.14	0.09	0.08	0.08	0.09	
H-CBG	Mean	8	2.83	2.17	0.77	0.42	0.45	0.48	0.07	83.33%
	SE		0.42	0.28	0.13	0.11	0.07	0.08	0.18	
Total	Mean	8	2.875	2.303	0.818	0.448	0.475	0.507	0.061	83.33%
	SE		0.265	0.194	0.095	0.071	0.051	0.055	0.097	

n = Number of samples, Na = Number of Different Alleles, Ne = Number of Effective Alleles, I = Shannon's Information Index, Ho = Observed Heterozygosity, He = Expected Heterozygosity, uHe = Unbiased Expected Heterozygosity, F = Fixation Index, PIC: polymorphism information content, Nei D = Nei's Genetic Distance, Nei I = Nei's Genetic Identity, Fst = inbreeding coefficient.
